# Supplementary material for: Analyzing information sharing behaviors during stance formation on COVID-19 vaccination among Japanese Twitter users
Source: PLoS One. 2024 Dec 31;19(12):e0299935. doi: 10.1371/journal.pone.0299935 (PMC11687874; doi:10.1371/journal.pone.0299935)
Supplement: Attachment — Submitted filename: response.pdf [file pone.0299935.s001.pdf]

Response to Reviewers

We sincerely appreciate the valuable feedback from Editor Ankit Gupta and the two anonymous reviewers. Our manuscript has been revised to address the questions and concerns raised by the reviewers. We await your reevaluation of our work. In this note, we outline the revisions made in response to your comments.

We first summarize the major revisions:

- Rephrase all sentences without "Vaccination stance classification" section (correspoding to Methods section) following PLOSONE's Ethical Publishing Practice [1]. We highlighted the rephrased sentence in black.
- Change all "stance change" and similar representations in the title and manuscript into "stance formation" based on the comment. Through our discussions, we have reached an agreement that the term "formation" was a more appropriate to represent actual situation.

In the following revision note, we provide a one-on-one response to each of the reviewers' comments and indicate how each issue is addressed in the revised manuscript. The new sentences were written in red and the deleted ones were written in strikethrough text. New figures or tables were attached a notation "(new)" at the last of their captions. Note that we have written the only rephrased sentences in black because almost all parts of our new manuscript was changed .

Unfortunately, we have had to cancel our plan to share our dataset in a public repository. Our original plan was to share a dataset that included tweet IDs and annotations showing different stances. However, getting permission from NTT Data, the source of our data, turned out to be a challenge. They are concerned that our dataset could go against their privacy policy. After considering this carefully, we have decided not to release the dataset for now.

Editor

Comment 1

[1] https://journals.plos.org/plosone/s/ethical-publishing-practice

Please ensure that your manuscript meets PLOS ONE's style requirements, including those for file naming. The PLOS ONE style templates can be found at

`https://journals.plos.org/plosone/s/file?id=wjVg/PLOSOne_formatting_sample_main_body.pdf` and

`https://journals.plos.org/plosone/s/file?id=ba62/PLOSOne_formatting_sample_title_authors_affiliations.pdf`

Answer 1
We revised some parts of our manuscript and other files which did not meet the requirements including file naming.

Revised parts

- Change the style of the title sentence case
- Change the symbol meaning indicating equal contributions to ¶
- Change the style of name of each section, subsection, and subsubsection into Bold style
- Change the name of supporting file into "S1_File.pdf"

Comment 2
In your Methods section, please include additional information about your dataset and ensure that you have included a statement specifying whether the collection and analysis method complied with the terms and conditions for the source of the data.

Answer 2
We have included additional information in dataset section about our dataset. Also, we have included a sentence in dataset section to make it sure that our collection and analysis method were in accordance with the terms and conditions of Twitter (X Corp.).

Revised parts

- Add an overview of our dataset construction process
- Add a consideration about bots and their impacts on our analyses

Comment 3 We noticed you have some minor occurrence of overlapping text with the following previous publication(s), which needs to be addressed: `https://ieeexplore.ieee.org/document/10068695`

In your revision ensure you cite all your sources (including your own works), and quote or rephrase any duplicated text outside the methods section. Further consideration is dependent on these concerns being addressed.

Answer 3
We cited our previous work and rephrase all duplicated representation outside the methods section ("Vaccination stance classification" section). Quantitatively, we ensured that in any paragraphs there were no n-gram where $n \geq 10$ using a tool to measure how many words a sentence were duplicated in each paragraph.

Comment 4 Please note that PLOS ONE has specific guidelines on code sharing for submissions in which author-generated code underpins the findings in the manuscript. In these cases, all author-generated code must be made available without restrictions upon publication of the work. Please review our guidelines at `https://journals.plos.org/plosone/s/materials-and-software-sharing#loc-sharing-code` and ensure that your code is shared in a way that follows best practice and facilitates reproducibility and reuse.

Answer 4 We shared all codes in GitHub: `https://github.com/rapnob/stance_formation`

Comment 5 Thank you for stating the following in the Acknowledgments Section of your manuscript: "This research was conducted as part of "COVID-19 AI & Simulation Project" run by Mitsubishi Research Institute commissioned by Cabinet Secretariat, JAPAN. The methods for analysis were developed with support from JST CREST Grant Number JPMJCR19A4 and JSPS KAKENHI Grant Number JP21H03445."

We note that you have provided funding information that is currently declared in your Funding Statement. However, funding information should not appear in the Acknowledgments section or other areas of your manuscript. We will only publish funding information present in the Funding Statement section of the online submission form.

Please remove any funding-related text from the manuscript and let us know how you would like to update your Funding Statement. Currently, your Funding Statement reads as follows: "This research was conducted as part of "COVID-19 AI & Simulation Project" run by Mitsubishi Research Institute commissioned by Cabinet Secretariat, JAPAN. The methods for analysis were developed with support from JST CREST Grant Number JPMJCR19A4

and JSPS KAKENHI Grant Number JP21H03445. The funders had no role in study design, data collection and analysis, decision to publish, or preparation of the manuscript."

Please include your amended statements within your cover letter; we will change the online submission form on your behalf.

Answer 5 We removed all funding-related text from Acknowledgements section and included the amended statements within our cover letter.

Comment 6 We noted in your submission details that a portion of your manuscript may have been presented or published elsewhere. [This paper is an extended version of our paper published in Diachronic Analysis of Users' Stances on COVID-19 Vaccination in Japan using Twitter, Proceedings of the 2022 IEEE/ACM International Conference on Advances in Social Networks Analysis and Mining (ASONAM), 2022, pp. 237-241. We have improved our classifier for stance detection, updated all experimental results, and added some new results.]

Please clarify whether this publication was peer-reviewed and formally published. If this work was previously peer-reviewed and published, in the cover letter please provide the reason that this work does not constitute dual publication and should be included in the current manuscript.

Answer 6 The paper mentioned above was originally published as a short paper (4 pages) in the ASONAM 2022 conference proceedings. It underwent a review and assessment by the program committee. Due to its brevity, we had to omit certain analyses and results, and the performance of our proposed stance classifier, as presented in the paper, was subpar, which affected the reliability of our findings.

In this paper, we have significantly improved the performance of the classifier and reconducted analyses to provide updated results. Consequently, it was necessary to include descriptions of these analyses and present the updated findings. Furthermore, in this revision, based on feedback from the editors and reviewers, we have updated and rephrased various sections to ensure that this work does not constitute dual publication.

Comment 7 We note that you have stated that you will provide repository information for your data at acceptance. Should your manuscript be accepted for publication, we will hold it until you provide the relevant accession numbers or DOIs necessary to access your data. If you wish to make changes to your Data Availability statement, please describe these changes in your cover letter and we will update your Data Availability statement to reflect the information you provide.

Answer 7 We have provided our dataset on the corresponding author's homepage. `https://www.tkl.iis.u-tokyo.ac.jp/~cs/PLOSONE-2023-stance-formation`

Comment 8 We note that Figure 8 in your submission contain copyrighted images. All PLOS content is published under the Creative Commons Attribution License (CC BY 4.0), which means that the manuscript, images, and Supporting Information files will be freely available online, and any third party is permitted to access, download, copy, distribute, and use these materials in any way, even commercially, with proper attribution. For more information, see our copyright guidelines: http://journals.plos.org/plosone/s/licenses-and-copyright.

Answer 8 Related to the Answer 3, Figure 8 in our manuscript in the current version is different from Figure 8 in the previous version of our work because we re-conducted all experiments and obtained different results. We believe Figure 8 does not contain copyrighted images. If our understanding is incorrect, I would appreciate it if you could inform me.

Reviewer 1

Comment 1
Please define the abbreviation 'COVID-19' in the first instance of its use.

Answer 1
We defined the abbreviation by introducing the below sentence in Introduction section. "the 2019 novel coronavirus disease (hereinafter called COVID-19) pandemic"

Comment 2
"... subsequent studies leveraged social media posts and focused on the 17 COVID-19 vaccination [6 – 9, 11, 12]" - a later study utilising Twitter as a database found that majority of negative sentiment tweets centered around the perceived coercive policies or vaccine mandates, superseding safety or efficacy concerns. This is relevant to mention (citation: `pubmed.ncbi.nlm.nih.gov/36146535`).

Answer 2 We added a reference to this study.
-5th paragraph in Introduction We added the below sentence into Introduction section.

" Currently, there is no consensus on common factors for negative sentiment toward vaccination, a later study found that majority of negative sentiment tweets were predominantly mentioned the coercive policies or vaccine mandates, rather than safety or efficacy concerns [citation] "

Comment 3 What is the theoretical framework for the study? Is there one? Why or why not?

Answer 3

As you said, there was a lack of explanation of our theoretical framework of this study. So, I added the explanation.

Revised parts

-4th paragraph in Introduction We added the below sentence into Introduction section.

"Several studies reported that SNS users' stances toward vaccination were influenced by the information they obtained on SNS, such as external links [citation, citation] or posts from other users [citation], resulting in wrong medical treatments like vaccine hesitancy or anti-social behaviours like hoarding. Based on the observations, we explored the relationship between Twitter users' stances and the external information sources such as news articles or other Twitter users which they referred to just before their stances were determined. To this end, we implemented a classifier for stance detection, which is an NLP task [citation] of predicting a stance (typically favor, against and none) of given text toward a certain target, and determined each Twitter user's stance toward COVID-19 vaccines. "

Comment 4 It is beneficial for the readers of the journal that a figure summarizing the methods and tweets selection process is provided.

Answer 4

We added an overview of the architecture of our classifier and tweets selection process for dataset construction.

Revised parts

-1st and 3rd paragraphs in Vaccination stance classification section

Comment 5 I am not sure if it is apt to title this paper as "Stance Shifts on COVID-19 Vaccination"; moreover, a rapid uptake of COVID-19 vaccination does not imply a stance shift per se but simply a natural result of the fact that vaccination for people aged 18 to 64 years only began around June 2021. This implies a receptive population rather than one that shifted from vaccine hesitant to vaccine receptive.

Answer 5 As you mentioned, stance shifts between pro-vaccine and anti-vaccine groups were uncommon in our study. To better reflect the focus of our analysis, we have adjusted the title from 'Stance Shifts on COVID-19 Vaccination' to 'Stance Formation on COVID-19

Vaccination,' emphasizing the examination of how initially neutral users formed their stance, and revised contents accordingly.

Revised parts
-Title -Almost all parts in which the phrase "stance shift/change" was used.

Comment 6 Related to the above, you can see that across the time periods, there was in fact no real stance shift and the anti-vaccine portion of tweets remained consistently small.

Answer 6 Similarly to Answer 5, the title and related parts were modified.

Comment 7 Frequent tweets are an important component in maintaining a following and therefore one limitation of this study is a lack of understanding about the representativeness of these findings. The tweets could originate from a very active few or could be more widespread. How should this be considered?

Answer 7
To limit the impact of very active users, we counted the same news articles (URLs) or the same user's posts shared by the same user repetitively only once, while we forgot to write that. So we added it. Additionally, although we found that anti-vaccine group has a tendency to post tweets more frequently than pro-vaccine in our recent study, the increase in the volume of anti-vaccine groups was very small as described in our manuscript. We will further investigate the reasons.

Revised parts
We added the below sentence in "What kinds of users were referred to by users who formed their stances?" subsubsection. in Analysis section.

"If one user referred to the same user multiple times during these processes, it was counted only once."

Comment 8 The main body of the manuscript is relatively thin, and it is recommended that the authors further dig into the existing data for in-depth analysis apart from framing this as a stance shift, which did not really materialise.

Answer 8 As we answered to the comment 5, we adjusted our focus to the stance formation of neutral users. To show the details of stance transition, we added a table displaying the number of people moved between stances and a discussion based on that table.

Revised parts 2nd paragraph in “Stance formations of users” subsection in Analysis section,

Comment 9 What is ”Full vaccination coverage” as defined in Figure 1? Is it simply receiving two doses? Please be clear.

Answer 9 We added the explanation.

Revised parts “ second dose of the vaccine (fully vaccinated at the time)” 2nd paragraph in Introduction section.

Comment 10 There is an extensive literature on these topics. Most importantly it is fundamental to take into consideration how political institutions/authorities have used Twitter during Covid and how this has also led to misunderstanding and misinformation in some cases.

Answer 10 In our case, we found an indications of their positive impacts. Taro Kono, Japan’s minister in charge of fighting COVID-19 at that time, often used SNSs like Twitter to propagate vaccine-related information or criticize misinformation about vaccines. As we mentioned in our manuscript, the occurrence of the word “Mr. Kono” indicated that people including those who were unsure to get vaccinated highly focused on his activity on SNS and the activity reduced their hesitancy in the vaccines. We wrote this analysis in the part of our wordcloud analysis.

Revised parts “ In July, many press reported that vaccination rate was found low and thus each prefecture’s governor requested people to get vaccinated, while those who hoped to get vaccinated could not make a reservation of vaccine vouchers due to troubles of reservation system at that time, resulting in their complaints to the governors and the emergence of the word “governor”. This trend was in accordance with the occurrence of the word “Mr. Kono”, who was a minister in charge of COVID-19 vaccinations and received a lot of criticism for his announcement of stopping additional procurement of vaccines in July. ”

Comment 11 Given that identifying bots is a major taproot in big data analytics, it is essential that some explanation be provided. What percentage of the data could be posted by bots?

Answer 11 We explored the impact of bot accounts and added the insights.

Revised parts ”Our investigation of the proportion of bot accounts revealed that the tweets posted from 4 major clients (iPhone, Android, iPad, and Web) accounted for 93.9% and the tweets posted from client applications including ”bot” in their name accounted for only about 1%, which meant that such accounts had only small impact on our analysis.“

Reviewer 2

Comment 1
The G7 nations should be explained.

Answer 1
We defined G7 nations clearly.

Revised parts
-Introduction 2nd paragraph 追加

Comment 2
Is the BERT Japanese version? which plm?

Answer 2
We specified that we used BERT in Japanese version. We also specified our model name of BERT.

Revised parts
-2nd paragraph in Experiments on vaccination stance classification subsection in ”Vaccination stance classification“ section We added the below sentence into Introduction section.

“ This Japanese version BERT was pre-trained on Japanese Wikipedia. We used NICT_BERT-base_JapaneseWikipedia_100K. ”

Comment 3 Please introduce the proposed model in detail to enable readers to understand the overall structure and advantages.

Answer 3
We added an overview figure of our classifier.

Revised parts

-1st paragraph in Text and graph-based stance classification subsection in ”Vaccination stance classification“ section

Comment 4 Some future works should be added, such as emotion fusion, topic modeling, and interpretable models and Chatgpt application.

Answer 4 We added the below sentence into Conclusion section.

Revised parts
-6th paragraph in Conclusion section

”For future work, some NLP techniques would be useful. A thematic analysis like LDA would brought about a fundamental knowledge about popular topics among the neutral-to-pro/anti users. Emotion fusion would provide us with in-depth understanding of stance formation process. Considering recent advances of chatbot services like chatGPT, we could use such services to annotate vaccine-related tweets. Also, interpretable models would offer insights into what types of tweets are labeled as each stance.“

Comment 5 Some references could be added: Topic detection and sentiment analysis in Twitter content related to COVID-19 from Brazil and the USA Emotions and topics expressed on Twitter during the COVID-19 pandemic in the United Kingdom: Comparative geolocation and text mining analysis Public opinion and sentiment before and at the beginning of COVID-19 vaccinations in Japan: Twitter analysis

Answer 5 We added the description of these studies.

Revised parts **-3rd paragraph in Related work section**

“ Alhuzali et al. [citation] combined sentiment analysis with geographical analysis by using tweets posted from each city in the United Kingdom to predict their sentiment labels using a deep learning model. Mønsted et al. [citation] used transfer learning to improve the performance of their stance classifier. They investigated propagation process of false information about vaccination on mutual mention/retweet network. Garcia et al. [citation] compared transition of COVID-19 related topics in Brazil with that of the USA in Twitter. They also used combined some deep learning model and embedding-based techniques. ”
