# Supplementary material for: Analyzing information sharing behaviors during stance formation on COVID-19 vaccination among Japanese Twitter users
Source: PLoS One. 2024 Dec 31;19(12):e0299935. doi: 10.1371/journal.pone.0299935 (PMC11687874; doi:10.1371/journal.pone.0299935)
Supplement: Attachment — Submitted filename: response.pdf [file pone.0299935.s002.pdf]

Response to Reviewers

We sincerely appreciate the valuable feedback from Editor Ankit Gupta and the two anonymous reviewers. Our manuscript has been revised to address the questions and concerns raised by the reviewers. We await your reevaluation of our work.

We have revised our paper in response to the comments from the editor and reviewers. Below, we detail the changes we made. Additionally, we have included an explanation of our user-filtering process, which was missing in the previous version. Note that this additional explanation does not affect our original results.

Editor

Comment 1

Please review your reference list to ensure that it is complete and correct. If you have cited papers that have been retracted, please include the rationale for doing so in the manuscript text, or remove these references and replace them with relevant current references. Any changes to the reference list should be mentioned in the rebuttal letter that accompanies your revised manuscript. If you need to cite a retracted article, indicate the article’ s retracted status in the References list and also include a citation and full reference for the retraction notice.

Answer 1

We rechecked our reference list and confirmed that it is complete and correct. None of them have been retracted.

1 Reviewer 1

Comment 1

I still have issues with the phrase ’stance formation’. I may choose to get vaccinated not because I accept the vaccine but simply because I deemed it to be necessary (or that it

has been strongly pushed for by the government). I may also have an attitude of ’vaccine acceptance’ but still not proceed to get vaccinated. In this study, the authors are only looking at people who appear to ’accept’ the vaccines. While vaccine acceptance and uptake are intricately linked, the terms are not interchangeable and have distinct constructs. I refer to the following systematic review and meta-analysis published in 2022: Mapping global acceptance and uptake of COVID-19 vaccination: A systematic review and meta-analysis (`https://www.nature.com/articles/s43856-022-00177-6`) which clearly shows that there is a difference between global COVID-19 vaccine acceptance (67.8%) and uptake (42.3%). While acceptance refers to an attitude, uptake refers to the actual act of receiving vaccination. The nuances should be explained.

Answer 1

We added a definition of stance formation in Introduction section, detailed explanations of each stance in Dataset section, and the limitations of our work in Conclusion section. In this paper, we defined both ’vaccine acceptance’ and ’vaccine uptake’ as the same label, Pro-vaccine, as showed in Table 3. In our annotation dataset, almost all of Pro-vaccine tweets (95.7%) reported the users got vaccinated (i.e., uptake) and only a small part of Pro-vaccine tweets (4.3%) presented their acceptance, which means that the users who accepted vaccines without getting vaccinated were very rare. Our analyses did not capture the users who did not showed ’vaccine acceptance’ while getting vaccinated.

As you noted, treating ’accept’ and ’update’ as distinct concepts would provide a more fine-grained analysis. For future work, we aim to conduct such analyses.

Comment 2

”In July, many press reported that vaccination rate was found to be low, and thus, each prefecture’ s governor requested people to get vaccinated, while those who hoped to get vaccinated could not make a reservation with a vaccine voucher due to trouble with the reservation system at that time, resulting in their complaints to governors and the emergence of the word “governor.” This trend was in accordance with the occurrence of the word “Mr. Kono,” who was a minister in charge of COVID-19 vaccinations and received a lot of criticism for his announcement of stopping additional procurement of vaccines in July” - at least a reference should be provided here.

Answer 2 We added some references with each event and changed all URL references to news articles into citations in the last second and third paragraphs in Analysis section.

Comment 3 ”Considering recent advances of chatbot services like ChatGPT, we could use such services to annotate vaccine-related tweets” - I understand this was suggested by the

other reviewer but I don't think this is specifically necessary and can be omitted.

Answer 3 We removed this sentence form Conclusion section.

Additional revise

We added an explanation of the user-filtering process in Dataset section. The target users of our analyses were those who have conducted information-sharing behaviours (retweet, mention, or quote) at least once and we extracted only such users, which was not explained in our previous version.
