# Supplementary material for: Analyzing information sharing behaviors during stance formation on COVID-19 vaccination among Japanese Twitter users
Source: PLoS One. 2024 Dec 31;19(12):e0299935. doi: 10.1371/journal.pone.0299935 (PMC11687874; doi:10.1371/journal.pone.0299935)
Supplement: Attachment — Submitted filename: letter.pdf [file pone.0299935.s003.pdf]

Response to Reviewers

Dear academic editors,

Our manuscript was previously submitted to PLOS ONE under the identifier PONE-D-23-22865R2. Although it was accepted in March of this year, we have found significant issues in our data processing pipeline before publication. We have since resolved these issues, updated the results along with the analysis, and resubmitted the manuscript under the identifier PONE-D-24-25295.

We have summarized our revisions in this letter. The overall result remains unchanged, but there are significant changes in Analysis section. We have corrected the data preprocessing and chi-squared test implementation and updated the results and description. Additionally, we have added supplemental explanations about the users' attributes and the site's categories. The main changes and corresponding subsections, subsubsections, and paragraphs are as follows.

Changes

Changes in "Stance formations of users" Although we wrote in the paper that users were selected based on the condition "limited to users who posted tweets in 10 out of 15 periods," the implementation did not follow this condition. When we actually applied this condition, the number of users was too small, so we relaxed the condition to "limited to users who posted tweets in at least 4 out of 5 months" and reanalyzed the data. (Line number: 365 – 368)

Changes in "Information-sharing behaviors associated with stance formation changing stances"

In "What kinds of users were referred to by users who formed their stances?" subsection (1st, 2nd, 4th, 5th paragraphs), and "What types of external sites were shared by users who determined their stances?" subsection (1st, 2nd paragraphs), we have revised several results as follows.

1. For the analysis of referred users and external sites, in addition to the chi-square results, we added graphs of the most frequently referred users and external sites. (Line number: 450 – 460, 524 – 547)

2. Some of the referred users and external sites have changed, so we have revised the analysis results accordingly, and Conclusion section has also been slightly modified. (There are no significant changes in the distribution of stances or the results of their formation) (Line number: 461 – 502, 548 – 553)

3. We revised the implementation of the chi-square test and conducted the test again. Additionally, we added frequency ranking graphs for comparison.

Others

1. All figures from figure 7 onwards have been recreated on the basis of new results.

2. Table 5 and 6 (definition tables for the attributes of referred user accounts and the categories of shared external sites) have been added.
