# Supplementary material for: Analyzing information sharing behaviors during stance formation on COVID-19 vaccination among Japanese Twitter users
Source: PLoS One. 2024 Dec 31;19(12):e0299935. doi: 10.1371/journal.pone.0299935 (PMC11687874; doi:10.1371/journal.pone.0299935)
Supplement: Attachment — Submitted filename: reb.pdf [file pone.0299935.s004.pdf]

Response to Reviewers

Dear academic editors,

We sincerely appreciate the valuable feedback from Editor Ankit Gupta and the anonymous reviewer. Our manuscript has been revised to address the questions and concerns raised by the reviewers. We await your reevaluation of our work.

We have summarized our revisions in this letter. We have also included additional descriptions and an explanation regarding our decision not to disclose certain information due to ethical considerations. The most recent changes are highlighted in blue. The responses to each comment are as follows. If changes were made, the detail of that changes are described; if no changes were made, the reasons are provided.

Changes

Changes in "Transition in polarization between vaccination stances"

Q: Why only anti-vaccine users remained active compared to pro-vaccine? Because they are bots or some other special case of users? I guess this is the thing that authors might be able to explain based on the data collected, but if it is not possible, that is fine.

Unfortunately, our further investigation revealed no rationale for their continuous activity. However, we examined 50 anti-vaccine accounts, and none were found to be bots. We have added this description. (Line number: 362 – 363, blue)

Changes in "Stance formations of users"

Line 365: This part is a bit confusing. Did you mean that you identified users who Tweeted at least four months out of five month of data collection window?

We have modified the description to clarify this point. (Line number: 369 – 370, blue)

Line 366: What is particular period here? specific month?

Line 367: ambiguity of ”particular period” makes me harder to understand the rest of the sentence.

We have divided each month into three periods as shown in the previous analyses (“Distribution of users’ stance” subsection). In this subsection, we clarified the aim of this subsection, noted that “period” has been defined previously, and included the definition again for clarity. (Line number: 371 – 374, blue)

Line 390: stances –> stance groups

We have modified the expression. (Line number: 398, blue)

Changes in “Information-sharing behaviors associated with stance formation changing stances”

Line 469-470: Is this provided in the material? If not, can it be shared in the main doc or supplementary section?

Line 491: Same for this – Is this provided in the supplementary material? If not, can it be shared in the main doc or supplementary section?

Line 538-539: It would be really great if I can see the raw data/title of these videos in supplementary section or sth.

Line 545-546: Also for this.

So my biggest comment is that ”why the authors only describe by text of the unseen data instead of at least indirectly providing them?”

Unfortunately, we cannot share the original tweet data publicly due to Twitter’s (X Corp.) restrictions on redistributing content to third parties (https://developer.twitter.com/en/developer-terms/agreement-and-policy). Given these restrictions and ethical concerns regarding privacy, we have only provided descriptions of the posts and included external sources. Identifying each user’s ideology and incorporating it into the paper could violate their privacy.
